# Supplementary material for: Mechanisms and therapeutic implications of RTA 408, an activator of Nrf2, in subarachnoid hemorrhage–induced delayed cerebral vasospasm and secondary brain injury
Source: PLoS One. 2020 Oct 5;15(10):e0240122. doi: 10.1371/journal.pone.0240122 (PMC7535038; doi:10.1371/journal.pone.0240122)
Supplement: S1 Data — (PPTX) [file pone.0240122.s001.pptx]

## Slide 1
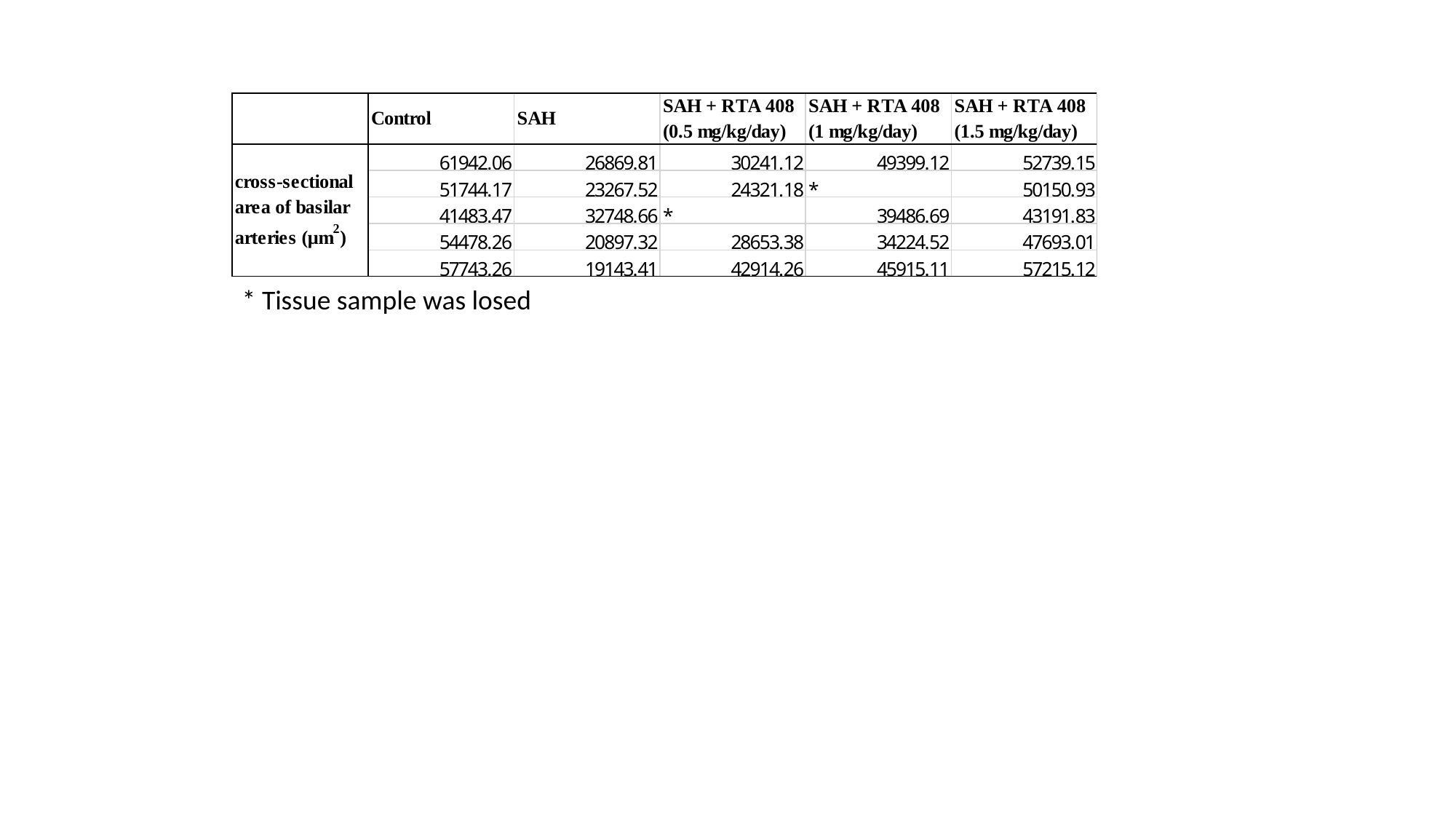

* Tissue sample was losed

## Slide 2
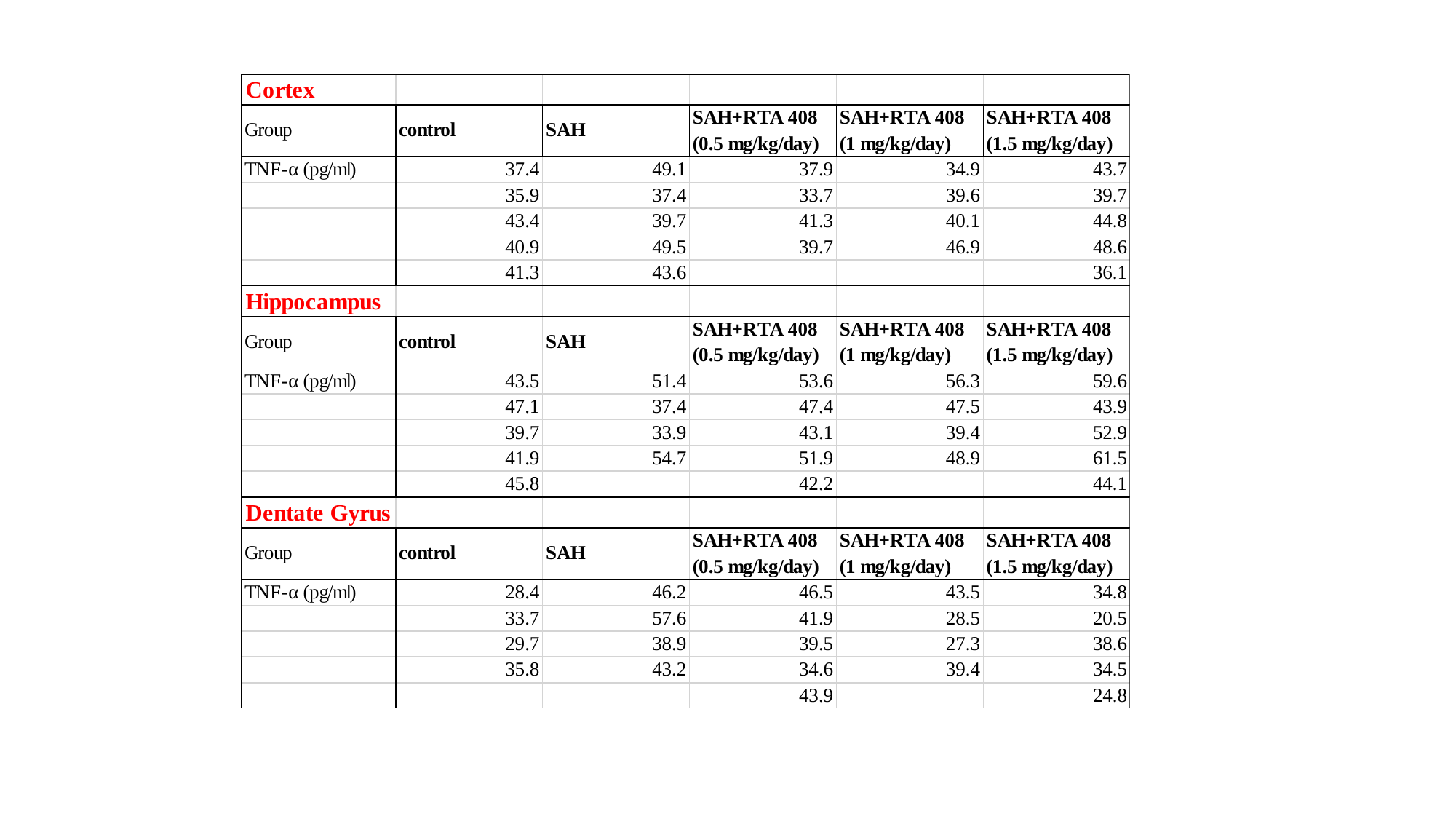

## Slide 3
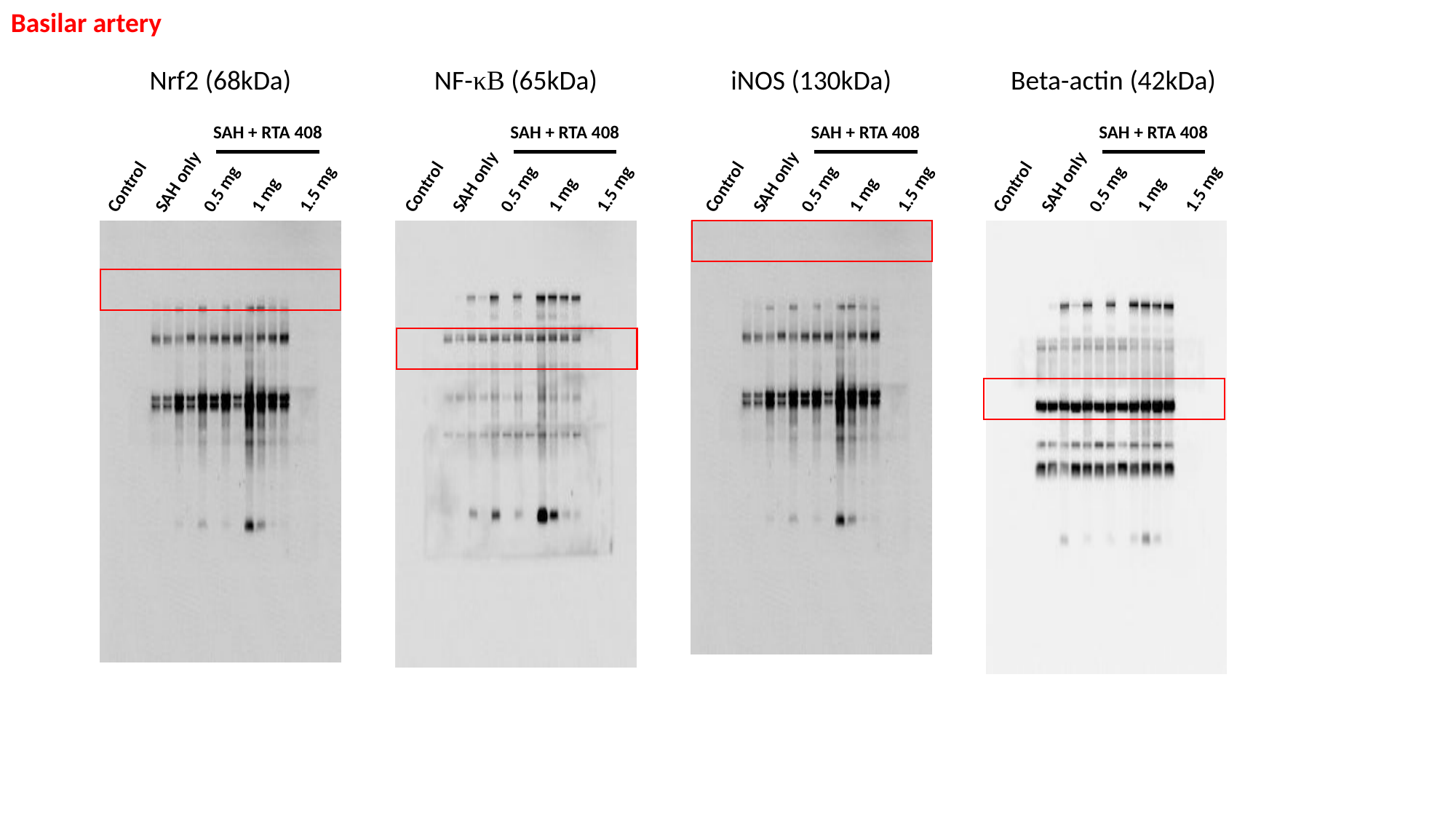

Basilar artery
Nrf2 (68kDa)
NF-κB (65kDa)
iNOS (130kDa)
Beta-actin (42kDa)
SAH + RTA 408
Control
SAH only
0.5 mg
1 mg
1.5 mg
SAH + RTA 408
Control
SAH only
0.5 mg
1 mg
1.5 mg
SAH + RTA 408
Control
SAH only
0.5 mg
1 mg
1.5 mg
SAH + RTA 408
Control
SAH only
0.5 mg
1 mg
1.5 mg

## Slide 4
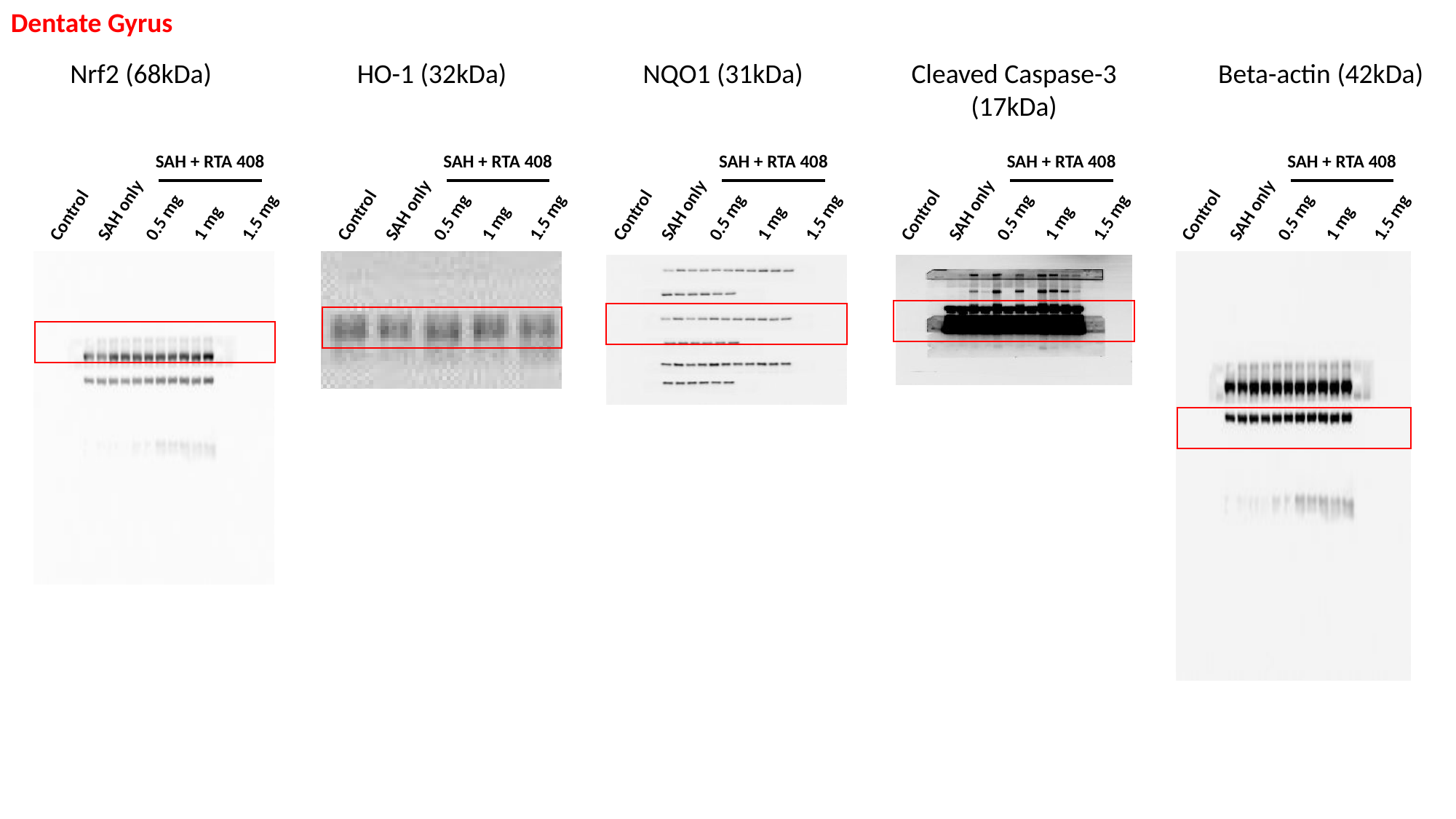

Dentate Gyrus
Nrf2 (68kDa)
HO-1 (32kDa)
NQO1 (31kDa)
Cleaved Caspase-3 (17kDa)
Beta-actin (42kDa)
SAH + RTA 408
Control
SAH only
0.5 mg
1 mg
1.5 mg
SAH + RTA 408
Control
SAH only
0.5 mg
1 mg
1.5 mg
SAH + RTA 408
Control
SAH only
0.5 mg
1 mg
1.5 mg
SAH + RTA 408
Control
SAH only
0.5 mg
1 mg
1.5 mg
SAH + RTA 408
Control
SAH only
0.5 mg
1 mg
1.5 mg

## Slide 5
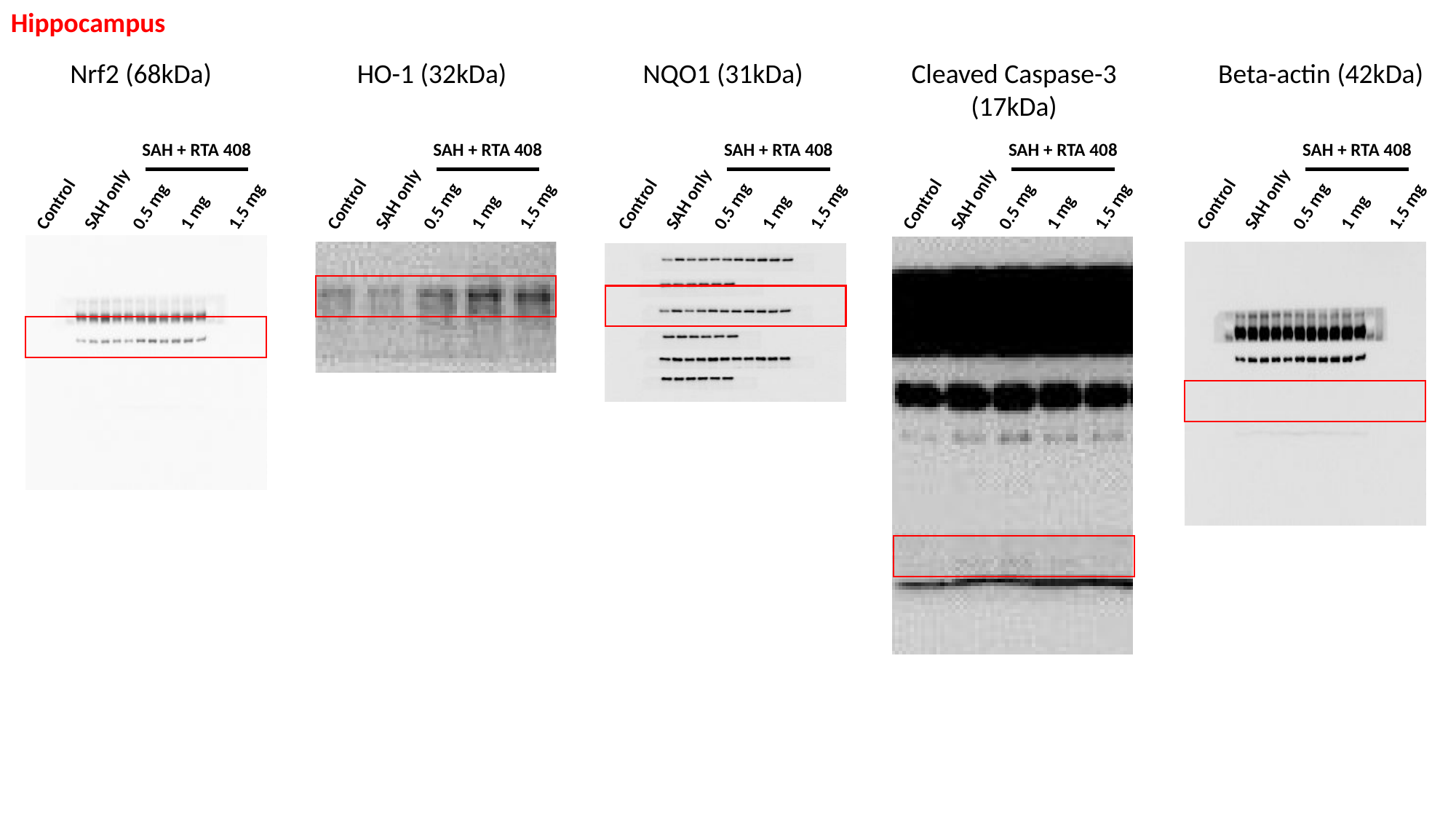

Hippocampus
Nrf2 (68kDa)
HO-1 (32kDa)
NQO1 (31kDa)
Cleaved Caspase-3 (17kDa)
Beta-actin (42kDa)
SAH + RTA 408
Control
SAH only
0.5 mg
1 mg
1.5 mg
SAH + RTA 408
Control
SAH only
0.5 mg
1 mg
1.5 mg
SAH + RTA 408
Control
SAH only
0.5 mg
1 mg
1.5 mg
SAH + RTA 408
Control
SAH only
0.5 mg
1 mg
1.5 mg
SAH + RTA 408
Control
SAH only
0.5 mg
1 mg
1.5 mg

## Slide 6
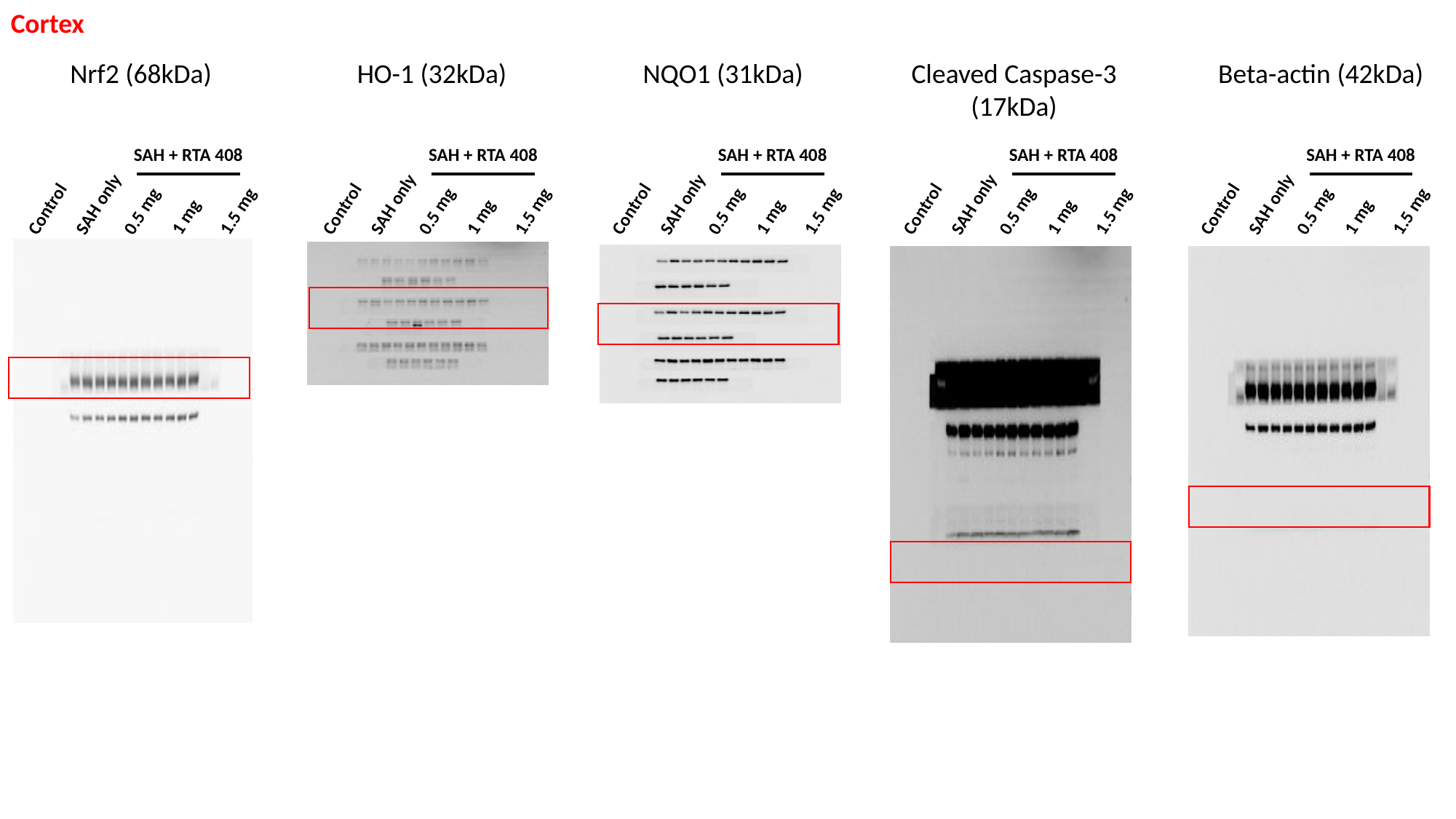

Cortex
Nrf2 (68kDa)
HO-1 (32kDa)
NQO1 (31kDa)
Cleaved Caspase-3 (17kDa)
Beta-actin (42kDa)
SAH + RTA 408
Control
SAH only
0.5 mg
1 mg
1.5 mg
SAH + RTA 408
Control
SAH only
0.5 mg
1 mg
1.5 mg
SAH + RTA 408
Control
SAH only
0.5 mg
1 mg
1.5 mg
SAH + RTA 408
Control
SAH only
0.5 mg
1 mg
1.5 mg
SAH + RTA 408
Control
SAH only
0.5 mg
1 mg
1.5 mg
