# Supplementary material for: Mechanisms and therapeutic implications of RTA 408, an activator of Nrf2, in subarachnoid hemorrhage–induced delayed cerebral vasospasm and secondary brain injury
Source: PLoS One. 2020 Oct 5;15(10):e0240122. doi: 10.1371/journal.pone.0240122 (PMC7535038; doi:10.1371/journal.pone.0240122)
Supplement: Attachment — Submitted filename: Response to reviewers comment.docx [file pone.0240122.s002.docx]

**Reply to reviewer's comment**

**Additional Editor Comments:**

**1.Please revise all your figures to make them look more professional.**

Answer :

I have revised all figures to make them look more professional.

**Reviewer 1**

1. Please ensure that your manuscript meets PLOS ONE's style requirements, including those for file naming. The PLOS ONE style templates can be found at

Answer:

The manuscript has meet PLOS ONE's style requirements as the PLOS ONE'S style templates.

2. At this time, we request that you please report additional details in your Methods section regarding animal care, as per our editorial guidelines

Answer:

We have been reported the additional details in Methods section regarding animal care such as the source of the rats, animal welfare, the method of euthanasia and postoperative care.

3. In the Methods section, please provide the product number and any lot numbers of the primary antibodies used in the Western blot analysis for your study.

Answer:

We have been provided the product numbers of the primary antibodies used in the Western blot analysis in this manuscript.

4. In the Methods section, please provide the source, product number and any lot numbers of the RTA-408 used in the animal experiments for your study.

Answer:

We have been provided the source, product number of the RTA 408 used in the animal experiments.

5. To comply with PLOS ONE submission guidelines, in your Methods section, please provide additional information regarding your statistical analyses, including the specific name and version of the software used.

Answer:

We have been provided the specific name and version of the software regarding the specific name and version of the software in the Methods section.

6. We noticed minor instances of text overlap with the following previous publication(s), which need to be addressed involves the (1) Introduction and (2) Results section (pages 8-9).

Answer:

We also noticed the text overlap with the previous publications. Although the previous publications are our own works, we have been rephrased and quoted the duplicate text to avoid plagiarism or self-plagiarism.

7. We note that you have indicated that data from this study are available upon request. PLOS only allows data to be available upon request if there are legal or ethical restrictions on sharing data publicly.

Answer:

Data from this study are available upon request and there are no restrictions on sharing data publicly. We will upload the data set necessary to replicate our study findings as Supporting Information files.

8. PLOS ONE now requires that authors provide the original uncropped and unadjusted images underlying all blot or gel results reported in a submission’s figures or Supporting Information files.

Answer:

We will provide the original uncropped and unadjusted images underlying all blot or gel results reported in a submission’s figures.

9. PLOS requires an ORCID iD for the corresponding author in Editorial Manager on papers submitted after December 6th, 2016.

Answer:

We have an ORCID iD for the corresponding author and that it is validated in Editorial Manager.

**Reviewer 2:**

1. There are some minor grammatical errors that should be corrected.

Answer:

The grammatical errors of the article have been edited and the editing certificate is attached.

2. How were the dosages of RTA 408 selected?

Answer:

In this experiment, Group 3-5 received experimental SAH with RTA 408 treatment (Intraperitoneal injection) 30 min after the first induction of SAH for 7 days following the first hemorrhage. The dosages of RTA 408 are 0.5 mg/kg/day, 1 mg/kg/day and 1.5 mg/kg/day. The dosages of RTA 408 were selected according to the previous experiments. Peng Han, et al. RTA-408 Protects Kidney from Ischemia-Reperfusion Injury in Mice via Activating Nrf2 and Downstream GSH Biosynthesis Gene. Oxid Med Cell Longev. 24 December 2017.

3. Did the authors examine Nrf2 nuclear translocation?

Answer：

The authors do not examine Nrf2 nuclear translocation, because this experimental design is for the study of downstream protein changes in the Nrf2 pathway caused by RTA 408 treatment. Despite the changes of several Nrf2 downstream of protein levels after RTA-408 treatment, the authors did not directly sought out a clear mechanism for the neuroprotective effects of RTA-408. Therefore, additional intervention studies including Nrf2 nuclear translocation are still needed to verify the links between RTA408 and Nrf2 and the consequences of SAH. This problem was addressed in the limitation section.

4. A limitations section should be provided.

Answer:

A limitation section has been provided.
